# Probiotic *Bifidobacterium bifidum* BGN4 supplementation modulates gut microbiome composition and reduces circulating zonulin, TNFα, and insulin in adults with excess adiposity: a randomized, double-blind, placebo-controlled trial

**DOI:** 10.1186/s12986-026-01124-1

**Published:** 2026-05-11

**Authors:** Ju-Won Choi, Hyeyoon Kim, Sung-Hee Ham, Ye-Jin Han, Sung-Eun Kim, Mi-Kyung Sung

**Affiliations:** Department of Food and Nutrition, Sookmyung Women’s University, Seoul, Republic of Korea

**Keywords:** Probiotic, Adiposity, Inflammation, Intestinal permeability, Gut microbiota

## Abstract

**Background:**

Obesity, characterized by excess body fat accumulation, is closely linked to the alteration of gut microbiota, which contribute to systemic inflammation. Probiotics intervention has emerged as a promising strategy favorably modulating gut microbiota composition in obese individuals accompanied by improvements in metabolic parameters. The objective of this study was to evaluate whether supplementation with *Bifidobacterium bifidum* BGN4 alters gut microbiota composition and to assess its associated effects on circulating zonulin, a marker of intestinal permeability, as well as metabolic parameters in individuals with excess adiposity.

**Methods:**

This randomized, double-blind, placebo-controlled trial involved 60 adults with excess body fat (body fat percentage ≥ 20% for males and ≥ 28% for females). A total of 60 adults were enrolled, and sex- and age-stratified randomization allocated 30 participants to each group. Participants received one capsule of *B. bifidum* BGN4 (9 × 10^9^ colony forming unit) or matched placebo for 8 weeks. Blood samples were analyzed for zonulin, TNFα, hs-CRP, glucose, insulin, lipid profiles, and total antioxidant capacity. Fecal samples were analyzed to determine alterations in gut microbiota composition.

**Results:**

A total of 58 participants, with 29 individuals in each group, successfully completed the 8-week intervention. Supplementation with *B. bifidum* BGN4 did not result in significant changes in BMI, body fat percentage, or the primary outcome, hs-CRP. In contrast, significant improvements were observed in serum zonulin (between-group differences: -1.61 ± 2.69 ng/mL), TNFα (between-group differences: -0.17 ± 0.26 pg/mL), and fasting insulin (between-group differences: -3.52 ± 10.25 μIU/mL). The probiotic intervention modulated the enrichment of several taxa, including *Bacteroides coprocola, Bifidobacterium catenulatum* group, *Lactiplantibacillus plantarum* group, and *Prevotella stercorea*. In addition, several microbial taxa demonstrated correlations with metabolic and inflammatory parameters. No adverse effects were observed, as indicated by stable liver enzyme concentrations, blood pressure, and gastrointestinal symptoms.

**Conclusion:**

The results indicate that *B. bifidum* BGN4 may serve as a preventive strategy for metabolic disorders in individuals with excess adiposity through the maintenance of gut microbial balance and intestinal barrier integrity thereby potentially mitigating inflammation and metabolic stress.

**Trial registration:**

Clinical trial registration number: KCT0010817. Date of registration: July 28, 2025.

**Supplementary Information:**

The online version contains supplementary material available at 10.1186/s12986-026-01124-1.

## Introduction

Obesity is defined as the abnormal or excess accumulation of body fat resulting from a chronic positive energy balance [1]. The global age-standardized prevalence of overweight and obesity among adults (aged ≥ 18 years) has steadily increased from 25% in 1990 to 43% in 2022, and over 50% of adults are expected to be overweight or obese by 2050 [1, 2]. Recently, the concept of metabolically healthy obesity (MHO) has emerged, referring to individuals with excess adiposity but without overt metabolic abnormalities [3]. However, growing evidence indicates that MHO is not a benign condition, as a considerable proportion of individuals transition to metabolically unhealthy states over time, thereby increasing the risk of chronic diseases. Consequently, obesity contributes to the growing burden of major noncommunicable diseases, including cancer, type 2 diabetes, and cardiovascular diseases, irrespective of metabolic status [4–6]. Accordingly, early management and intervention are warranted even in individuals who appear metabolically healthy.

Numerous studies have shown that obesity is associated with gut microbial alterations, characterized by reduced microbial diversity and shifts in key bacterial taxa, such as *Akkermansia muciniphila* and *Bifidobacterium longum* [7, 8]. These compositional shifts impair intestinal barrier integrity and increase gut permeability [9–11]. This dysfunction of the gut barrier facilitates the translocation of endotoxins, such as lipopolysaccharides (LPS), into the systemic circulation [12–14]. The presence of circulating endotoxins triggers chronic low-grade inflammation and disrupts insulin signaling, ultimately contributing to the progression of metabolic disturbances in obesity [13, 15, 16]. In addition, these metabolic alterations intensify inflammatory processes, leading to sustained metabolic stress and a higher risk of obesity-related diseases [17]. In humans, obesity is associated with a reduction in short-chain fatty acid (SCFA)-producing bacteria such as *Oscillospira* and *Christensenella* and an increase in proinflammatory microbial taxa, which may contribute to immune dysregulation and metabolic impairment [18–21]. Given the mechanistic links between impaired intestinal barrier function, inflammation, and metabolic dysfunction, modulating the gut microbiota has emerged as a promising target for obesity-related interventions, with growing evidence supporting the role of probiotics in restoring microbial balance and mitigating obesity-associated inflammation [22, 23].

A recent meta-analysis evaluating the efficacy of probiotic supplementation in obese individuals reported a significant mean reduction in body weight, waist circumference, and abdominal fat area [24]. The primary probiotic strains used in this meta-analysis were *Bifidobacterium* spp. and *Lactobacillus spp.* These beneficial bacterial strains are known to enhance SCFAs production, strengthen gut barrier integrity, modulate bile acid metabolism, and suppress inflammation by inhibiting endotoxin-mediated signaling pathways. This increases energy expenditure and improves obesity-related metabolic outcomes [25].

*Bifidobacterium bifidum* BGN4 (*B. bifidum* BGN4) was originally isolated from a healthy breast-fed infant’s fecal sample in 1996. The immunomodulatory effects of *B. bifidum* BGN4, particularly its ability to suppress the production of proinflammatory cytokines, have been well documented in animal and human studies [26–28]. However, it remains unclear whether these effects are also relevant in metabolically healthy individuals with excessive adiposity, a condition characterized by subtle inflammatory imbalance despite the absence of overt metabolic dysfunction. Based on these findings, we hypothesized that *B. bifidum* BGN4 supplementation would modulate gut microbiota composition and be associated with changes in serum zonulin, marker of intestinal permeability, as well as in inflammatory and metabolic parameters, including high-sensitivity C-reactive protein (hs-CRP), TNF-α, and insulin, in individuals with excessive adiposity. Therefore, we conducted a randomized, double-blind, placebo-controlled human intervention study to evaluate whether *B. bifidum* BGN4 supplementation could improve gut permeability, metabolic parameters, and the gut microbiota composition in individuals with excessive adiposity.

## Material & methods

### Participant recruitment and eligibility criteria

Participants were recruited from April to May 2024 through advertisement posters on campus and local community centers and postings on university online platforms. The inclusion criteria were (1) men and women aged 19 to 64 years and (2) body fat percentage above the normal range (≥ 20% for males and ≥ 28% for females) [29, 30]. Participants were excluded if they (1) had a diagnosis or history of serious or chronic diseases (e.g., malignancies, diabetes, inflammatory bowel disease, liver, or cardiac dysfunction), or experienced any relevant medical history including prior surgeries; (2) used medications or functional foods specially intended to reduce body fat within the last 1 month; (3) used probiotics, prebiotics, or synbiotics in the last 1 month; (4) received antibiotic treatment within the past 3 months; (5) reported habitual alcohol consumption exceeding 20 g/day for women or 30 g/day for men; (6) were allergic to probiotics or excipients; and (7) were pregnant or lactating during the study period. Volunteers who had participated in a similar clinical trial within the past month were also excluded. Those who met all the inclusion criteria and none of the exclusion criteria provided written informed consent and were enrolled in the study. This study was approved by the Institutional Review Board of Sookmyung Women’s University, Korea (approval number: SMWU-2402-BR-126–01) and was registered with the Clinical Research Information Service (registration number: KCT0010817, https://cris.nih.go.kr/cris/search/detailSearch.do?seq=29833&search_page=L&search_lang=E&class_yn=).

### Study design and randomization

A randomized, double-blind, placebo-controlled, parallel-group intervention trial was conducted in accordance with the Consolidated Standards of Reporting Trials (CONSORT) guidelines. Randomization was performed using sex- and age-stratified block randomization. All subjects were categorized into six strata defined by sex (male or female) and age group (19–34, 35–50, and 51–64 years). Within each stratum, a separate block randomization procedure assigned participants in a 1:1 ratio to the probiotic (*n* = 30) or placebo group (*n* = 30). A third person, who was not associated with this study, conducted randomization and assigned participants to interventions. All subjects completed two study visits at baseline (week 0) and the end of the intervention (week 8), following an overnight fast of ≥ 8 h. In addition, one online survey was conducted at week 4. Questionnaires on alcohol consumption, smoking status, and physical activity were administered for baseline assessment at week 0. Anthropometric measurements and safety parameter measurements were conducted at two study visits. Gastrointestinal symptoms were recorded at weeks 4 and 8. All subjects completed a three-day dietary record (2 weekdays and 1 weekend day) to assess their typical diets using the 24-h recall method. Dietary intake of energy, carbohydrates, proteins, fats, fatty acids, and fiber was calculated using the Computer Aided Nutritional analysis program (CAN-Pro 6.0, Korean Nutrition Society, Seoul, Korea). Fecal samples were self-collected in Mediclin® All-PP Stool/Sputum Specimen Container (Daehan Scientific, Seoul, Korea) and submitted to the research staff immediately upon arrival at the study site. All blood and fecal samples were stored at −80 °C until analysis.

### Probiotic intervention and compliance assessment

All supplements were provided by BIFIDO Co., Ltd. (Hongcheon, Republic of Korea). A total of 58 probiotic or placebo capsules were provided to each participant, individually sealed in blister packs, and contained in white packages. One probiotic capsule contained 405 mg of *B. bifidum* BGN4 and 45 mg of maltodextrin (total 450 mg per capsule), providing 9.0 × 10⁹ CFU per dose. Viable cell counts were confirmed at baseline by CFU enumeration. One placebo capsule contained 446.868 mg of maltodextrin, along with trace amounts of colorants and flavoring agents (total 450 mg per capsule). The products were stored at 2–8 °C both prior to distribution and throughout the intervention period. Both probiotic and placebo supplements were matched in appearance, color, and weight, and labeling was carried out by two research assistants from an independent laboratory not involved in the study. All participants were instructed to take one capsule per day with water, preferably after a meal, for a total of 8 weeks. The products were refrigerated both prior to distribution and throughout the intervention period. Compliance was assessed by counting the number of unused capsules returned at the end of the study period (week 8). Participants who consumed 80% or more of the capsules provided during the 8-week intervention period were considered compliant.

### Anthropometric measurements

Body weight and height were measured to the nearest 0.01 kg and 0.1 cm using an automatic digital stadiometer and scale (DS-102; Dong Sahn Jenix Co., Ltd., Seoul, Korea). The participants were barefoot, wore light clothing during the measurement, and stood upright on the platform in a freestanding position, allowing the device to simultaneously record both weight and height. Body mass index (BMI) was calculated as weight (kg) divided by height squared (m^2^). Body composition, including body fat percentage, was assessed using a bioelectrical impedance analyzer (InBody 620; Inbody Co., Ltd., Seoul, Korea). The participants were instructed to remove any metal accessories and stand upright on the foot electrodes with their bare feet placed according to marked positions. They were also asked to hold the hand electrodes with their arms slightly abducted from the torso. After entering age, sex, height, and weight into the device, participants maintained their posture for approximately 1–2 min until the analysis was completed. The measurements were performed under fasting conditions.

### Lifestyle factors assessment

Lifestyle factors, including smoking, drinking, and physical activity, were collected at baseline (week 0) using a self-administered questionnaire. Participants were asked to report their smoking and drinking status by indicating whether they were current users, former users, or had never smoked or consumed alcohol. Alcohol consumption was assessed using a structured questionnaire that captured beverage type, typical serving size (mL), and average frequency of intake. Weekly alcohol intake (units/week) was then estimated using the following formula:

$$unit/week = \frac{Volume \left [mL\right] \times ABV \times 0.789}{14} \times frequency\ per\ week$$

where 0.789 represents the specific gravity of ethanol (g/mL), and one unit was defined as 14 g of pure alcohol, according to both U.S. and Korean public health guidelines[31, 32].

Physical activity levels were assessed using a self-administered questionnaire adapted from the physical activity classification system provided in the CAN-Pro program developed by the Korean Nutrition Society. Participants were classified into three categories—high, moderate, or low—based on frequency, intensity, and duration of aerobics or daily activities.

### Blood biomarkers analyses

Serum zonulin, an intestinal permeability marker, was assessed using a colorimetric human zonulin ELISA kit (NBP2-80,305; Novus Biologicals, LLC, Centennial, CO, USA). Serum TNFα was quantified using a high-sensitivity ELISA kit (HSTA00E; R&D Systems, Minneapolis, MN, USA). Serum TAC was determined using a colorimetric TAC assay kit (EEA024; Thermo Fisher Scientific, Waltham, MA, USA). Other biomarkers including hs-CRP, fasting glucose, insulin, lipid profiles, aspartate aminotransferase (AST), and alanine aminotransferase (ALT) were analyzed by the Seegene Medical Foundation (Seoul, Republic of Korea), a certified clinical laboratory. The concentration of hs-CRP was measured by turbidimetric immunoassay (TIA) on a Cobas 8000 c702 module (Roche Diagnostics, Mannheim, Germany) using the CRPHS reagent (Roche Diagnostics, Mannheim, Germany). Briefly, glucose concentration was measured by the ultraviolet (UV) method using an automated analyzer (Labospect 008AS; Hitachi, Japan) with the Qualigent GLU reagent (30,167,002; Sekisui Medical Co., Ltd., Tokyo, Japan), and insulin concentration was quantified by electrochemiluminescence immunoassay (ECLIA) using the Cobas 8000 e801 module (Roche Diagnostics, Mannheim, Germany) with the Elecsys Insulin reagent (12,017,547,122; Roche Diagnostics, Mannheim, Germany). The homeostasis model assessment of insulin resistance (HOMA-IR) was calculated using the following formula [33]:

$$HOMA\mathrm{-}IR = \text{fasting insulin }[\mu IU/mL] \times \text{fasting glucose }[mg/dL] / 405$$

Lipid profiles, including triglycerides (TG), total cholesterol, low-density lipoprotein cholesterol (LDL-C), and high-density lipoprotein cholesterol (HDL-C), were analyzed by the enzymatic method with the Labospect 008AS analyzer (Hitachi, Tokyo, Japan) using the corresponding enzymatic reagents (Qualigent series; Sekisui Medical Co., Ltd., Tokyo, Japan). For safety assessment, AST and ALT were determined by the UV method using an automated analyzer (Labospect 008AS; Hitachi, Tokyo, Japan) with the Qualigent AST or ALT reagent (38,499,000, 38,556,000; Sekisui Medical Co., Ltd., Tokyo, Japan).

### Safety parameters and gastrointestinal symptoms assessment

Safety outcomes, including systolic blood pressure (SBP), diastolic blood pressure (DBP), and pulse rate, were measured at baseline and at the end of the 8-week intervention using an automated blood pressure monitor (Omron HEM-7142T2, Kyoto, Japan). Measurements were taken after the participants had rested for at least 5 min in a seated position. Gastrointestinal (GI) symptoms were assessed via self-administered online surveys at weeks 4 and 8. Participants recorded the severity of five GI symptoms including flatulence, abdominal bloating, abdominal pain, bowel rumbling, and bowel cramps on a 4-point scale (with 0 indicating no symptoms, 1 mild symptoms, 2 moderate symptoms, and 3 severe symptoms).

### Fecal DNA extraction

Fecal DNA was extracted from 120 mg of stool using the microMag® Nucleic Acid Extraction System (NEXT&BIO, Seoul, Republic of Korea). DNA concentration and purity were assessed using the Epoch® Microplate Reader and Gen5 software version 3.0 (BioTek Instruments, Inc., Winooski, VT, USA). The concentration of extracted DNA was adjusted to 10 ng/μL using elution buffer and stored at −80 °C deep freezer until further analysis.

### Quantification of *B.bifidum* BGN4 by qPCR

Quantification of *B. bifidum* BGN4 was performed using real-time (quantitative polymerase chain reaction (qPCR) on a 7500 Fast Real-Time PCR system (Applied Biosystems, Foster City, CA, USA) with qPCRBIO SyGreen Mix Lo-Rox (PCR Biosystems Ltd., London, UK). Each 20 μL reaction mixture consisted of 10 μL of SyGreen mix, 1 μL each of forward and reverse primers, 3 μL of buffer, and 5 μL of DNA template. The thermal cycling conditions included an initial denaturation at 95 °C for 2 min (ramp rate 3.66 °C/s), followed by 40 cycles of denaturation at 95 °C for 10 s (3.66 °C/s) and annealing/extension at 60 °C for 30 s (2.2 °C/s). This was followed by a melt curve analysis consisting of 95 °C for 5 s (3.66 °C/s), 65 °C for 1 min (2.2 °C/s), and 97 °C for 1 s (0.2 °C/s). *B. bifidum* BGN4 was quantified by using *B. bifidum* BGN4 strain-specific primers (forward: 5′-GGA CGC GCT GAT CGT CTC GGT GAC GAC CG-3′; reverse: 5′-TGA GCG ACA GCT GGC ACG TGA ACA TCG AGG C-3′) [34]. For normalization, the universal bacterial 16S rRNA gene was used as an internal reference, amplified with primers targeting the V3 region (338F: 5′-ACT CCT ACG GGA GGC AGC-3′ and a 518R-derived reverse primer: 5′-GTA TTA CCG CGG CTG CTG GCA C-3′).

### Microbiota profiling based on 16 s rRNA gene sequencing

Fecal DNA quality was initially assessed during downstream sequence processing, and samples failing quality control were re-processed according to standard procedures. PCR amplification was performed using fusion primers targeting the V3–V4 regions of the bacterial 16S rRNA gene. The fusion primers consisted of Illumina adapter sequences, index sequences, Nextera consensus sequences, and the target-specific primers 341 F and 805R. Amplification was conducted under the following conditions: initial denaturation at 95 °C for 3 min, followed by 25 cycles of denaturation at 95 °C for 30 s, annealing at 55 °C for 30 s, and extension at 72 °C for 30 s, with a final elongation at 72 °C for 5 min.

PCR products were confirmed by electrophoresis on a 1% agarose gel and visualized using a Gel Doc system (Bio-Rad, Hercules, CA, USA). Amplicons were purified using magnetic bead-based cleanup, pooled at equimolar concentrations, and short non-target fragments were removed using the ProNex® Size-Selective Purification System (Promega, Madison, WI, USA). Library quality and concentration were assessed using the QuantiFluor® dsDNA System (Promega, Madison, WI, USA). Sequencing was performed on the Illumina MiSeq platform (Illumina, San Diego, CA, USA) at CJ Bioscience (Seoul, Republic of Korea), following the manufacturer’s instructions.

Raw sequencing reads were processed using the EzBioCloud 16S-based Microbiome Taxonomic Profiling (MTP) pipeline (CJ Bioscience, Seoul, Republic of Korea). Low-quality reads (Q < 25) were filtered using Trimmomatic (version 0.32) and paired-end reads were merged using VSEARCH. Primer sequences were trimmed, and non-16S rRNA sequences were removed using hidden Markov model-based filtering. Chimeric sequences were identified and removed using reference-based chimera detection. Taxonomic assignment was performed using the EzBioCloud database. For identification of taxa differentially enriched following the intervention, linear discriminant analysis effect size (LefSe) was applied within the probiotic group to compare baseline and post-intervention samples and identify taxonomic biomarkers associated with probiotic supplementation.

### Sample size calculation

Sample size estimation was based on serum hs-CRP as the primary endpoint for obesity-related systemic inflammation. Effect size estimation was conducted using data from four previously published randomized controlled trials that reported between-group differences in hs-CRP following probiotic or related interventions. For each study, the mean difference and corresponding standard deviation of hs-CRP changes between the intervention and control groups were extracted and used to calculate Cohen’s *d* effect size.

The required sample size was determined using G*Power software (version 3.1.9.7), which is commonly used in clinical trial planning. A power analysis was performed, assuming a two-sided test with 80% power and a significance level of 0.05. The estimated sample sizes per group from the four studies were 19, 22, 27, and 28. Based on the median of these estimates (*n* = 24), accounting for a 25% dropout rate, the final target sample size was set at 30 participants per group (total *n* = 60).

### Statistical analysis

All statistical analyses were performed using SAS software (version 9.4, SAS Institute Inc., Cary, NC, USA). Most data followed a normal distribution and are therefore presented as mean ± standard deviation (SD). Normality of the data was assessed using the Shapiro–Wilk test. Differences between the two groups were evaluated using independent-samples *t-*tests or Wilcoxon rank-sum tests, depending on the distribution of the data. Within-group changes from baseline to week 8 were analyzed using paired *t*-tests or Wilcoxon signed-rank tests. Correlation analyses were conducted using Spearman’s correlation coefficients for continuous variables or biserial correlation coefficients for associations between continuous and binary variables. Differences were considered statistically significant at *P* < 0.05.

## Results

### Baseline characteristics of enrolled subjects

**Fig. 1:**
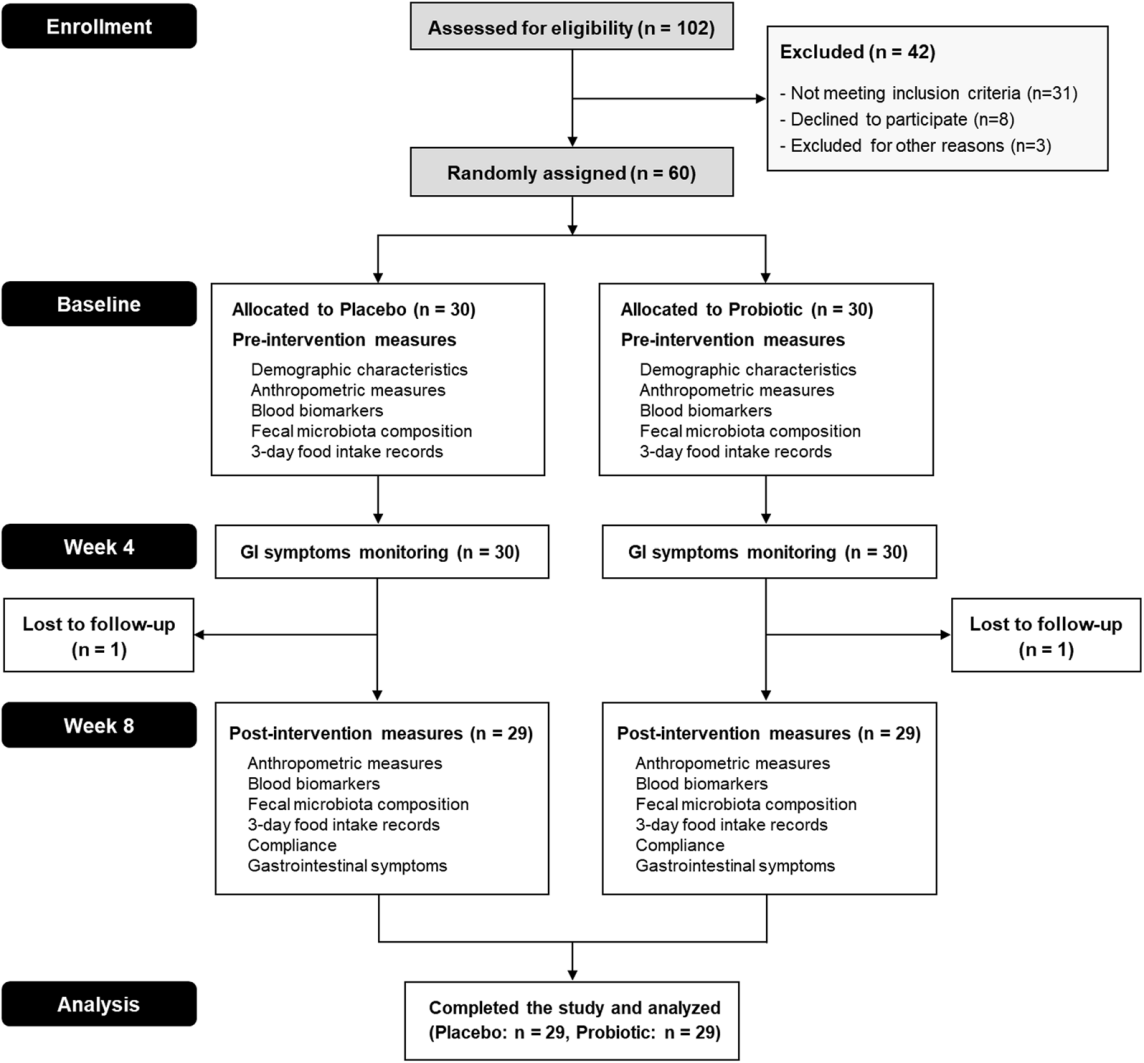
CONSORT flow diagram of participant enrollment, allocation, and analysis​. GI, gastrointestinal

A total of 102 volunteers were screened for eligibility, of whom 42 were excluded (Fig. 1). Among these, 31 did not meet the inclusion criteria, 8 declined participations (4 participants had personal scheduling conflicts; 4 patients reported poor physical condition), and 3 were excluded for other reasons, such as loss of contact prior to intervention. Sixty participants were randomized to either the placebo group (*n* = 30) or the probiotic group (*n* = 30) using stratified randomization based on age and sex. One participant from each group was lost to follow-up during the intervention, resulting in 29 participants in each group being included in the analysis. No statistically significant differences were observed between the placebo and probiotic groups in baseline demographic and anthropometric characteristics, including age, sex, and lifestyle-related variables such as smoking, drinking, and physical activity, except for BMI (Table 1). The daily nutrient intake at baseline and week 8 showed no significant changes (Supplementary Table S1).

**Table 1:** Baseline demographic and anthropometric characteristics of the study participants (n = 58)^1^

	Placebo (n = 29)	Probiotic (n = 29)			*P* value
Age, y	25 [24, 37]	24 [21, 40]			*0.41*
Gender					1.00
Male, n (%)	10 (34.5)	10 (34.5)			
Female, n (%)	19 (65.5)	19 (65.5)			
Height, cm	163.3 ±7.1	164.0±9.7			0.72
Weight, kg	59.5 [56.5, 66.1]	71.2 [58.6, 77.4]			0.07
Body fat, %	32.1 [29.5, 34.5]	32.6 [28.5, 35.8]			0.37
Body mass index, kg/m^2^	23.3 [21.8, 24.8]	25.9 [23.4, 28.6]			0.04
Drinking status					0.38
Non-drinker, n (%)	7 (24.1)	10 (34.5)			
Past-drinker, n (%)	0 (0.0)	1 (3.4)			
Drinker, n (%)	22 (75.9)	19 (62.1)			
Alcohol consumption, unit/week	1.2 [0.2, 2.3]	0.3 [0.0, 2.5]			0.33
Smoking status					0.20
Non-smoker, n (%)	25 (86.2)	22 (75.9)			
Past-smoker, n (%)	0 (0.0)	3 (10.3)			
Current smoker, n (%)	4 (13.8)	4 (13.8)			
Physical activity level					0.55
Low, n (%)	8 (27.6)	9 (31.0)			
Moderate, n (%)	15 (51.7)	17 (58.6)			
High, n (%)	6 (20.7)	3 (10.4)			

*P* values were determined using independent-samples *t* tests, Wilcoxon rank-sum tests, or Chi-square tests. These tests were applied to normally distributed variables expressed as mean ± SD, skewed variables expressed as median [interquartile range], and categorical variables expressed as n (%), respectively

### Anthropometric parameters and blood biomarkers

#### Anthropometric parameters

After the 8-week intervention, neither group showed significant changes in body weight and BMI, and there were no significant differences in weight changes between the groups (Table 2). Similarly, no significant difference in body fat percentage was observed between the two groups.

#### Circulating zonulin

**Table 2:** Effects of *B. bifidum* BGN4 probiotic supplementation on anthropometric measures and blood biomarkers (n = 58)^1^

	Placebo (n = 29)			Probiotic (n = 29)			Between-group difference	
	Baseline	Week 8	*P* value ^2^	Baseline	Week 8	*P* value ^2^	Change	*P* value ^3^
Body weight, kg	64.50 ± 2.76	64.27 ± 2.78	0.45	70.66 ± 2.81	71.08 ± 2.89	0.75	0.32 ± 2.22	0.69
Body mass index, kg/m^2^	23.96 ± 3.84	23.87 ± 3.87	0.36	26.12 ± 3.62^*^	26.07 ± 3.52^*^	0.69	0.05 ± 0.78	0.72
Body fat, %	31.80 ± 0.87	30.84 ± 0.99	0.36	33.22 ± 1.32^*^	32.54 ± 1.16^*^	0.67	0.28 ± 2.12	0.44
Fasting glucose, mg/dL	89.70 ± 11.20	87.0 ± 9.00	0.08	90.2 ± 10.50	89.3 ± 11.60	0.50	1.76 ± 11.51	0.37
Fasting insulin, μU/mL	8.04 ± 5.11	9.24 ± 6.10	0.14	12.68 ± 8.26^*^	10.35 ± 6.78	0.08	−3.52 ± 10.25	0.04
HOMA-IR	1.85 ± 1.32	2.01 ± 1.40	0.31	2.85 ± 1.97^*^	2.28 ± 1.55	0.09	−0.73 ± 2.46	0.06
TNFα, pg/mL	0.46 ± 0.17	0.53 ± 0.19	0.0009	0.56 ± 0.26	0.46 ± 0.18	0.0089	−0.17 ± 0.26	0.0006
hs-CRP, mg/L	0.98 ± 1.38	0.86 ± 1.46	0.95	0.64 ± 0.63	0.65 ± 0.61	0.73	0.13 ± 1.37	0.60
Zonulin, ng/mL	3.35 ± 0.42	4.42 ± 0.48	0.02	3.32 ± 1.34	2.77 ± 0.91^*^	0.07	−1.61 ± 2.69	0.0037
TAC, μmol/L	12.85 ± 0.69	13.47 ± 0.68	0.51	13.12 ± 0.48	12.54 ± 0.60	0.34	−1.20 ± 7.06	0.28
Total cholesterol, mg/dL	198.59 ± 25.22	201.28 ± 26.53	0.43	199.62 ± 33.96	199.41 ± 37.24	0.94	−2.90 ± 25.64	0.51
HDL cholesterol, mg/dL	59.28 ± 13.18	58.86 ± 12.35	0.72	54.72 ± 10.56	55.48 ± 10.58	0.50	1.17 ± 8.54	0.75
LDL cholesterol, mg/dL	117.62 ± 24.05	119.55 ± 27.20	0.47	120.38 ± 29.20	121.14 ± 30.91	0.78	−1.17 ± 21.79	0.69
TG, mg/dL	84.86 ± 43.60	88.03 ± 41.76	0.60	107.14 ± 83.46	97.45 ± 55.22	0.29	−12.86 ± 64.74	0.26

^1^ Values are expressed as mean ± SD. Abbreviations used include: HOMA-IR, homeostatic model assessment of insulin resistance; TNFα, tumor necrosis factor α; hs-CRP, high-sensitivity C-reactive protein; TAC, total antioxidant capacity

^2^ For within-group comparisons, paired-samples t-tests were employed for normally distributed data, while Wilcoxon signed-rank tests were utilized for skewed data

^3^ Differences between groups in changes from baseline were evaluated using independent-samples t-tests for normally distributed data and Wilcoxon rank-sum tests for skewed data. Asterisks (*) indicate significant differences between groups at the same time point, with *P* < 0.05

**Fig. 2:**
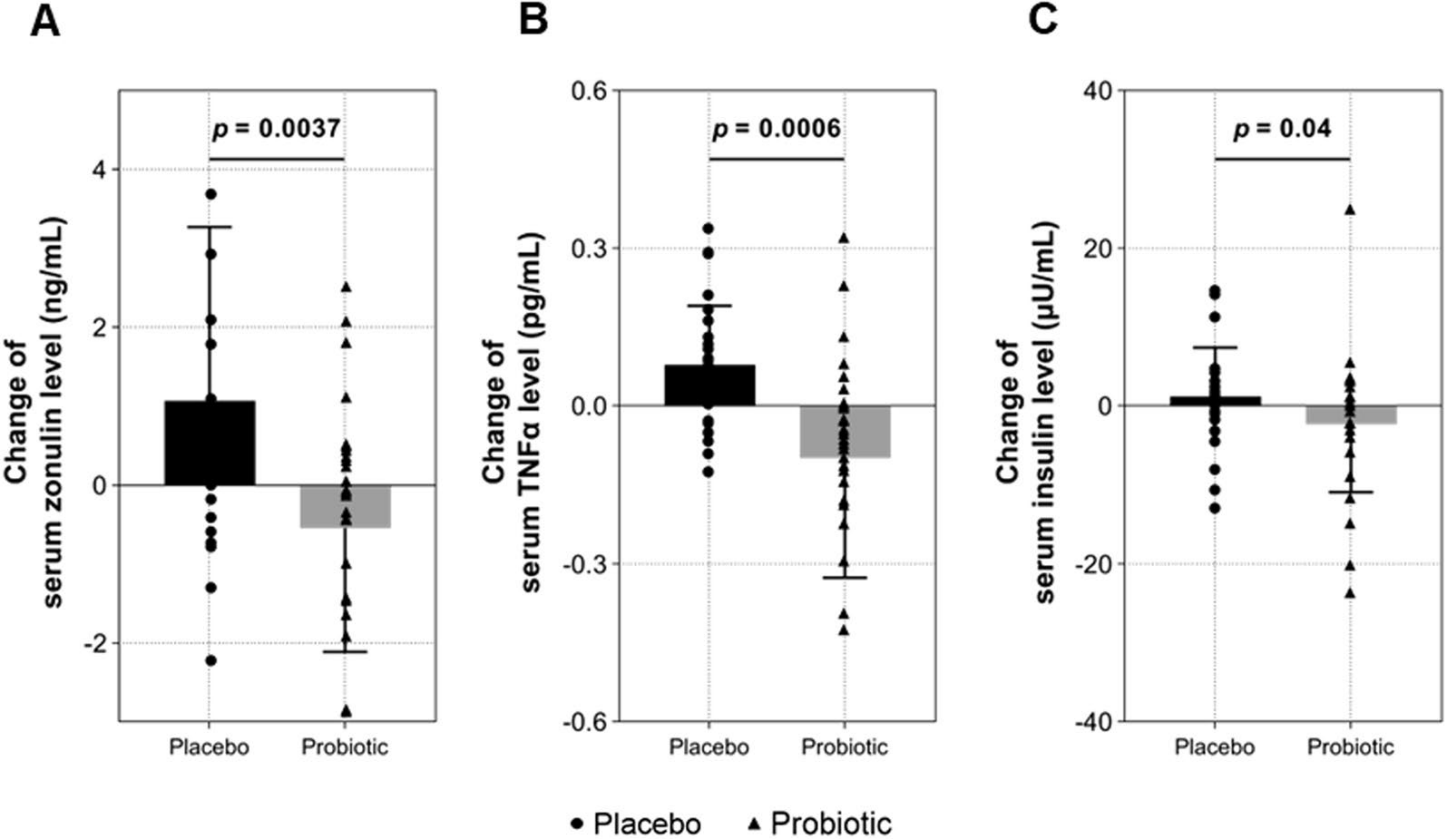
Alterations in serum concentrations of (**A**) fasting insulin, (**B**) TNFα, and (**C**) zonulin between groups (n = 29). These changes were determined by subtracting baseline values from those at week 8. The data are expressed as mean ± SD. For the purpose of between-group comparisons, Wilcoxon rank-sum tests were employed

The mean concentration of zonulin significantly increased in the placebo group and tended to decrease in the probiotic group compared with baseline, with a significant between-group difference in the change (−1.61 ± 2.69 ng/mL, *P* = 0.0037; Fig. 2A).

#### Inflammatory parameters

Among the inflammatory markers, TNFα concentration was significantly increased in the placebo group and significantly decreased in the probiotic group (−0.17 ± 0.26 pg/mL, *P* = 0.0006; Fig. 2B). No significant change was observed in hs-CRP levels, the primary outcome, between the groups following the intervention.

#### Metabolic parameters

Fasting glucose concentration did not show any significant change within or between the groups. On the other hand, fasting insulin concentration decreased by 2.33 ± 8.56 μIU/mL in the probiotic group and increased by 1.20 ± 6.19 μIU/mL in the placebo group, resulting in a statistically significant between-group difference (−3.52 ± 10.25 μIU/mL, *P* = 0.04; Fig. 2C). The changes in HOMA-IR also tended to be lower in the probiotic group than in the placebo group. No significant changes in lipid profiles (total cholesterol, HDL-C, LDL-C, TG) and TAC were observed between the two groups.

#### Correlation between body composition and blood biomarkers

Given that the study participants were BMI-defined overweight yet relatively young, serum biomarkers were largely within normal reference ranges. Nevertheless, the present study found that anthropometric measures and multiple biomarkers were significantly associated. Body weight was positively correlated with concentrations of fasting glucose, fasting insulin, HOMA-IR, TNFα, TAC, and TG. In addition, the concentration of glucose was positively associated with hs-CRP concentration, and TNFα concentration was positively correlated with zonulin concentration (Supplementary Figure S1).

### Safety parameters and gastrointestinal symptoms outcomes

Biochemical safety parameters, including AST and ALT concentrations, did not show significant changes within or between the groups over the 8-week intervention period (Table 3). Similar results were observed for systolic and diastolic blood pressures and pulse rate.

**Table 3:** Effects of *B. bifidum* BGN4 probiotic supplementation on safety parameters (n = 58)^1^

	Placebo (n = 29)							Probiotic (n = 29)							Between-group difference			
	Baseline			Week 8			*P* value^2^	Baseline			Week 8			*P* value^2^	Change			*P* value^3^
Systolic BP, mmHg	123.10 ± 21.50			119.80 ± 15.70			0.23	123.10 ± 25.13			119.83 ± 15.68			0.33	1.07 ± 17.42			0.76
Diastolic BP, mmHg	81.60 ± 10.70			81.10 ± 8.70			0.72	85.00 ± 11.60			82.80 ± 11.00			0.10	−1.79 ± 9.49			0.35
Pulse, bpm	84.80 ± 13.10			83.30 ± 13.90			0.97	83.80 ± 13.90			80.80 ± 13.20			0.36	−1.45 ± 25.91			0.59
AST, U/L	22.59 ± 3.51			21.59 ± 6.06			0.07	28.38 ± 35.25			22.52 ± 7.26			0.88	−4.86 ± 34.43			0.35
ALT, U/L	17.86 ± 6.99			17.14 ± 6.55			0.48	22.69 ± 17.85			21.79 ± 13.73			0.88	−0.17 ± 18.42			0.99

^1^ Values are expressed as mean ± SD. BP, blood pressure; AST, aspartate aminotransferase; ALT, alanine aminotransferase

^2^ For within-group comparisons, paired-samples t-tests were employed for normally distributed data, while Wilcoxon signed-rank tests were utilized for skewed data

^3^ To evaluate the differences between groups in changes from baseline, independent-samples t-tests were employed for normally distributed data, while Wilcoxon rank-sum tests were utilized for skewed data

**Fig. 3:**
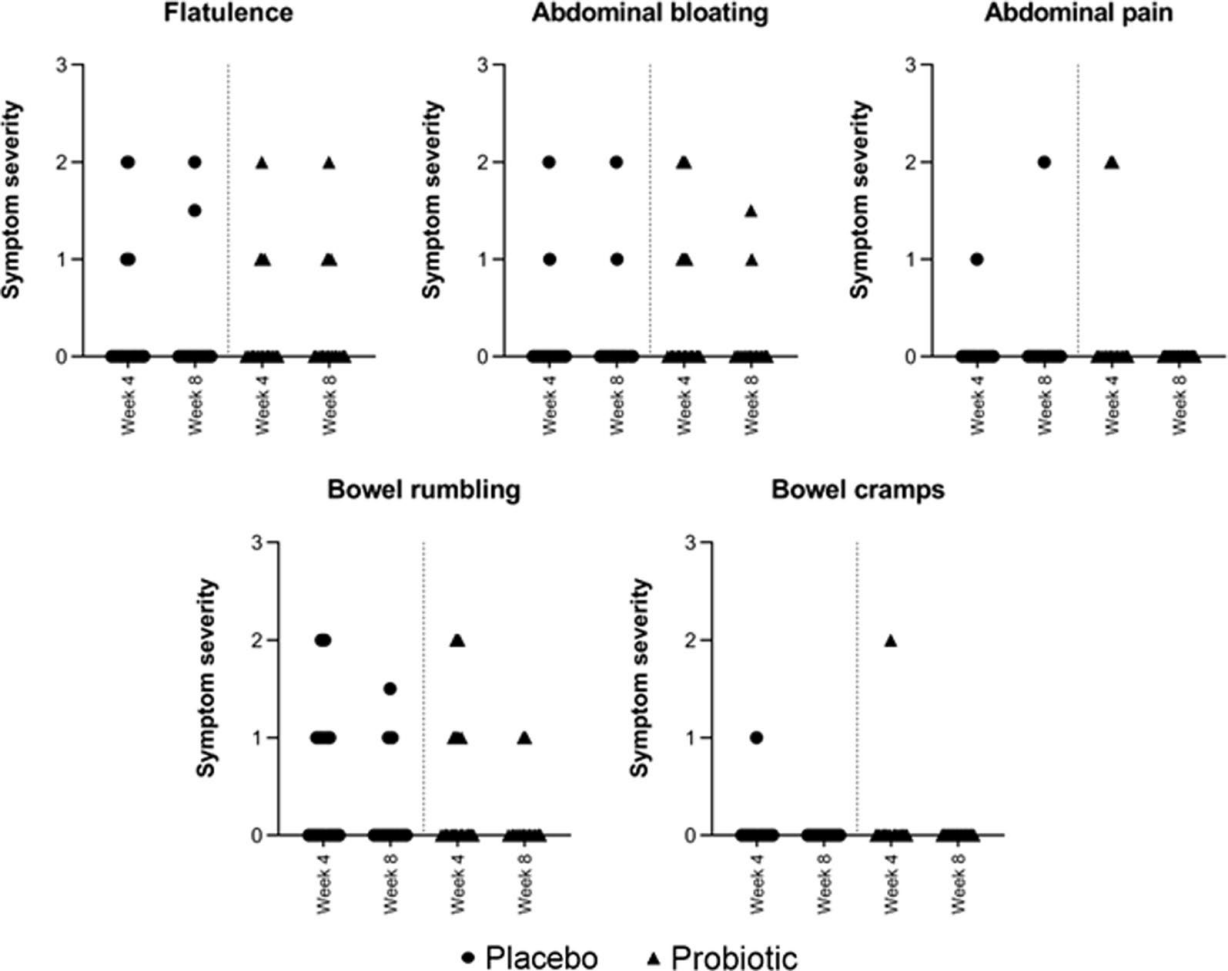
Severity of gastrointestinal (GI) symptoms in the placebo and probiotic groups (n = 29). Each dot represents the GI symptom severity score of an individual participant (0: no symptoms, 1: mild, 2: moderate, 3: severe). Wilcoxon signed-rank tests or Wilcoxon rank-sum tests were employed for within-group or between-group comparisons, respectively. No significant differences were observed in GI symptom severity scores following the intervention (*P* < 0.05). GI, gastrointestinal

Gastrointestinal symptoms, including abdominal pain, bloating, flatulence, bowel rumbling, and cramps, did not differ significantly between the probiotic and placebo groups after 8 weeks of intervention (Fig. 3). No significant within-group changes were observed compared with the baseline in either group.

### Fecal microbiota composition

#### B. bifidum BGN4 quantification

**Fig. 4:**
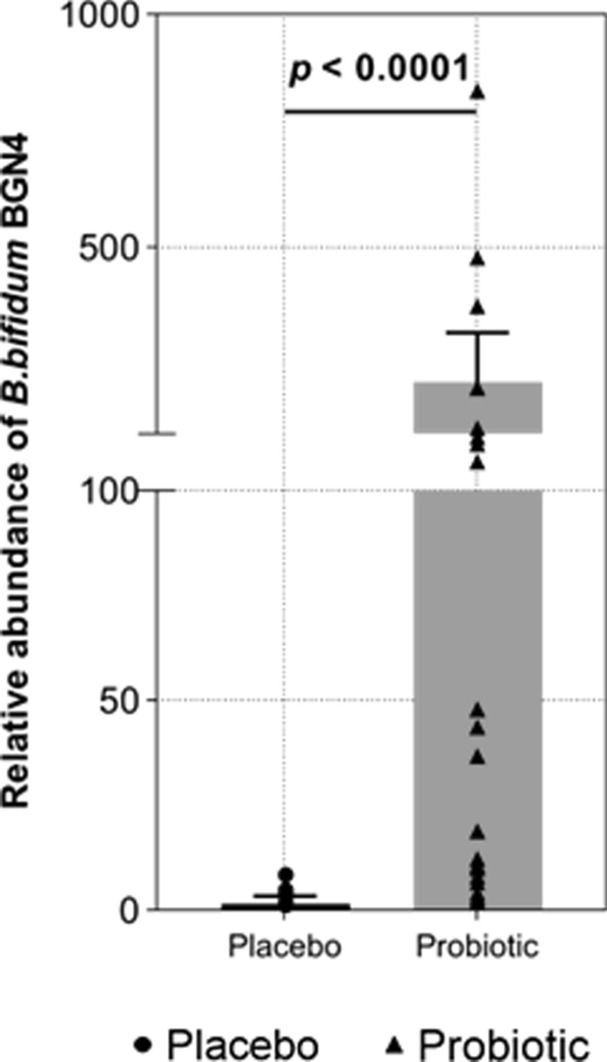
The relative abundance of *B. bifidum* BGN4 in fecal samples following the intervention (n = 29). DNA abundance of *B.bifidum* BGN4 were quantified by qPCR and normalized to bacterial 16S rRNA gene. Data are calculated using 2^−ΔΔCt^ method. The data are expressed as mean ± SEM. Wilcoxon rank-sum test was used for between-group comparison. *B. bifidum*, *Bifidobacterium bifidum*; SEM, standard error of the mean

After 8 weeks of intervention, the relative abundance of *B. bifidum* BGN4 was significantly elevated in the probiotic group compared to that in the placebo group (*P* < 0.0001; Fig. 4). The mean expression level was 101.1 ± 42.9 in the probiotic group and normalized to 1.0 ± 0.16 in the placebo group, indicating that *B. bifidum* BGN4 expression was approximately 101-fold higher after supplementation.

#### Changes in relative abundance

**Fig. 5:**
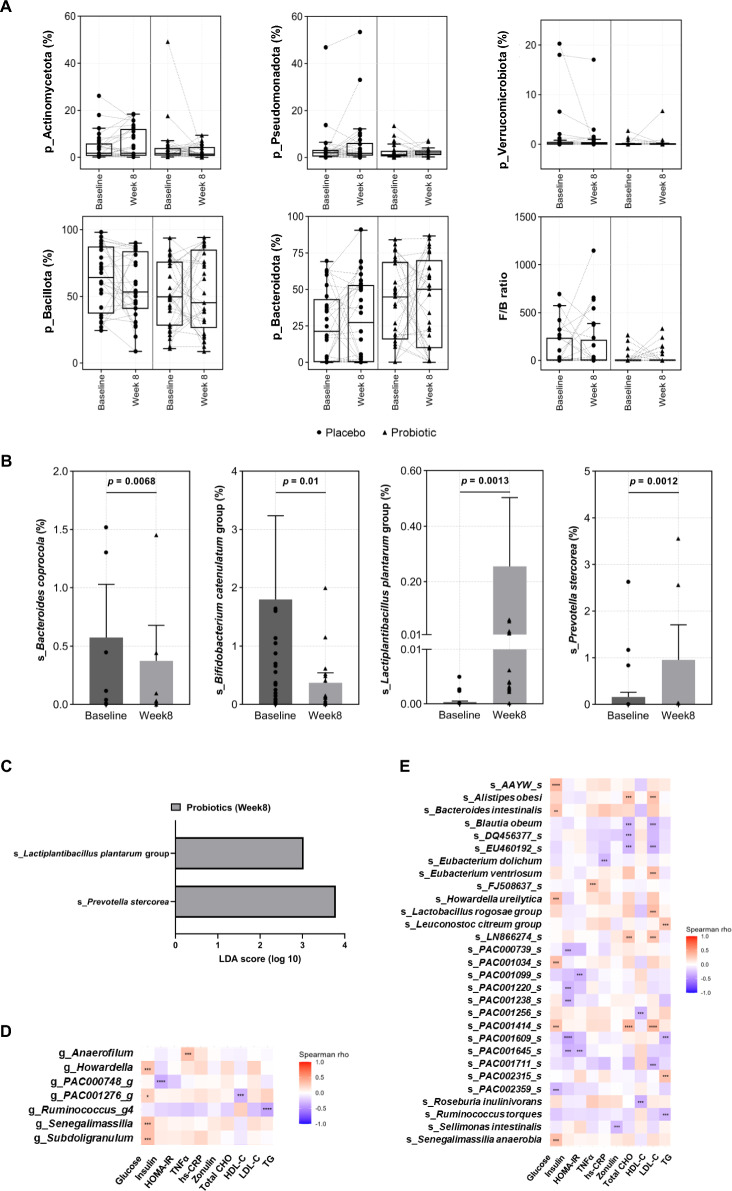
Effects of *B. bifidum* BGN4 on fecal microbiota and associations with blood biomarkers. (**A**) Differences in the relative abundance of the five most prevalent bacterial phyla and the Firmicutes-to-Bacteroidetes (F/B) ratio. Paired samples from the same individual are represented by a connecting black line. (**B**) The relative abundance of four bacterial taxa significantly altered in the probiotic group. Data are presented as mean ± SEM. Wilcoxon signed-rank tests were used for within-group comparisons. (**C**) Linear Discriminant Analysis (LDA) Effect Size (LEfSe) plots illustrating the differences in microbial taxa between baseline and week 8 in the probiotic group. (**D**) A heatmap illustrating Spearman’s correlation coefficients between blood biomarkers and bacterial taxa at genus levels, and (**E**) at the species levels. Taxonomic levels are indicated using prefixes (e.g., “p” for phylum, “g” for genus, and “s” for species). F/B, Firmicutes-to-Bacteroidetes; LDA, linear discriminant analysis; LEfSe, linear discriminant analysis effect size

Figure 5A shows the relative abundance of the five most dominant bacterial phyla such as Actinomycetota, Psuedomonadota, Verrucomicrobiota, Bacillota, and Bacteroidota and the Bacillota-to-Bacteroidota ratio (formerly referred to as the Firmicutes-to-Bacteroidetes ratio, F/B ratio) at baseline and after 8 weeks of intervention in the placebo and probiotic groups. No significant within-group or between-group differences were observed in the relative abundance of these phyla or the F/B ratio after the intervention. The relative abundance of *Bacteroides coprocola* (*B. coprocola*; *P* = 0.0068) and *Bifidobacterium catenulatum* group (*P* = 0.01) significantly decreased after the intervention group (Fig. 5B). Concurrently, the abundance of *Lactiplantibacillus plantarum* group (*L. plantarum* group; *P* = 0.0013) and *Prevotella stercorea* (*P. stercorea*; *P* = 0.0012) significantly increased from baseline to week 8. LEfSe analysis further revealed that the *L. plantarum* group and *P. stercorea* were the discriminant taxa significantly enriched by probiotic supplementation (Fig. 5C). No significant microbiome diversity differences were detected between the groups (Supplementary Fig. 2 and Supplementary Table 1).

### Correlation between fecal microbiota and blood biomarkers

Spearman’s correlation analysis was conducted to assess associations between bacterial taxa and blood biomarkers across all participants, in order to evaluate overall relationships between microbial taxa and metabolic markers (Fig. 5D). At the genus level, *Anaerofilum* was positively correlated with TNFα (*P* = 0.0004). *Howardella*, *Senegalimassila*, and *Subdoligranulum* were negatively associated with glucose (*Howardella*, *P* = 0.0005; *Senegalimassila*, *P* = 0.0003; *Subdoligranulum*,* P* = 0.0003). *PAC000748_g* was positively correlated with insulin (*P* < 0.0001). *PAC001276_g* was positively associated with glucose (*P* = 0.0101) and negatively associated with HDL (*P* = 0.0004). *Ruminococcus_g4* was negatively associated with TG (*P* < 0.0001). At the species level, glucose was positively associated with several taxa including *AAYW_s* (*P* < 0.0001), *Bacteroides intestinalis* (P = 0.0011), *Howardella ureilytica* (P = 0.0001), *PAC001034_s* (*P* = 0.0002), and *Senegalimassilia anaerobia* (*P* = 0.0003) and negatively associated with *PAC002359_s* (*P* = 0.0008). Insulin was negatively associated with *PAC000739_s* (*P* = 0.0008), *PAC001220_s* (*P* = 0.0005), *PAC001238_s* (*P* = 0.0003), *PAC001609_s* (*P* < 0.0001), and *PAC001645_s* (*P* = 0.0003). Similarly, HOMA-IR was negatively associated with *PAC001099_s* (*P* = 0.0002) and *PAC001645_s* (*P* = 0.0007). TNFα was positively associated with *FJ508637_s* (*P* = 0.0007), whereas hs-CRP was negatively associated with *Eubacterium dolichum* (*P* = 0.0006). Zonulin was negatively associated with *Sellimonas intestinalis* (*P* = 0.0008). Total cholesterol and LDL-C were positively associated with *Alistipes obesi* (total cholesterol, *P* = 0.0007; LDL-C, *P* = 0.0007) and *LN866274_s* (total cholesterol, *P* = 0.0006; LDL-C, *P* = 0.0007) and negatively associated with *Blautia obeum* (total cholesterol, *P* = 0.0008; LDL-C, *P* = 0.0006) and EU460192_s (total cholesterol, *P* = 0.0006; LDL-C, *P* = 0.0003). HDL-C was negatively associated with *PAC001256_s* (*P* = 0.0007) and *Roseburia inulinivorans* (*P* = 0.0002). TG was positively associated with *Leuconostoc citreum* group (*P* = 0.0008) and *PAC002315_s* (*P* = 0.0004), and negatively associated with *PAC001609_s* (*P* = 0.0008) and *Ruminococcus torques* (*P* = 0.0008).

## Discussion

In the present randomized, double-blind, placebo-controlled study, we investigated the effects of an 8-week supplementation with *B. bifidum* BGN4 on markers of intestinal permeability, inflammation, metabolic stress and gut microbiota composition in adults with excessive body fat.

Obesity is known to be accompanied by structural and functional alterations in the intestinal environment, including changes in gut microbiota composition, which have been linked to systemic inflammation and metabolic disturbances [35–37]. In this context, probiotic supplementation has emerged as a promising strategy to modulate gut environment and mitigate obesity-associated metabolic risk [38, 39]. Probiotics exert beneficial effects on microbial composition, gut barrier function, and host metabolism, thereby lowering obesity-induced metabolic endotoxemia and the associated inflammatory and metabolic disturbances [40, 41]. Bifidobacteria are recognized as early-life colonizers that play significant roles in the development of the immune system and metabolic processes [42]. Among these, *B. bifidum* BGN4 has demonstrated immunomodulatory, barrier-protective, and anti-obesity effects in preclinical models [40, 43]. Based on this evidence, we examined whether supplementation with *B. bifidum* BGN4 could mitigate systemic inflammation by modulating gut microbiota and enhancing intestinal permeability in adults with excess body fat.

In the present study, the primary changes in metabolic and inflammatory indices observed following *B. bifidum* BGN4 supplementation were decreases in circulating TNFα and fasting insulin concentrations. These blood biomarkers are widely recognized as early indicators of metabolic stress and low-grade inflammation [44]. Importantly, such systemic changes may be associated with alterations in gut barrier integrity and gut microbiota composition [45].

Consistent with this notion, *B. bifidum* BGN4 supplementation led to a significant reduction in the concentration of serum zonulin compared to the placebo group, indicating improved intestinal barrier function. Zonulin is an endogenous regulator of epithelial tight junctions that controls paracellular permeability, allowing selective absorption of nutrients and electrolytes while preventing the translocation of pathogens and toxic substances into the systemic circulation [46]. Increased intestinal permeability has been reported in obesity and insulin-resistant states and has been associated with obesity-related metabolic inflammation [9, 47]. Thus, the observed decrease in serum zonulin provides a plausible mechanistic link between gut barrier improvement and the concomitant reductions in circulating inflammatory and metabolic biomarkers. Recent studies further support the role of gut microbiota in regulating intestinal permeability and host inflammatory responses [23, 45].

Suggesting a coordinated improvement in gut barrier integrity, *B. bifidum* BGN4 supplementation also altered the gut microbiota composition by increasing beneficial taxa, including the *L. plantarum* group and *P. stercorea*. Previous studies have reported that *L. plantarum* upregulates tight junction proteins, including claudin-1, occludin, and ZO-1, and may enhance gut barrier function, in part through modulation of the zonulin pathway [48–50]. Therefore, the increase in *L. plantarum* group observed in this study might be associated with serum zonulin levels, a marker of intestinal permeability. Although *P. stercorea* is among the dominant *Prevotella* species in healthy individuals, its functional role remains incompletely understood [51]. Members of the *Prevotella* genus are known to produce SCFAs that contribute to mucosal immune homeostasis [52]. In addition, the reduced abundance of *B. coprocola*, a species associated with impaired glycemic control, suggests a microbial shift toward a metabolically favorable profile following BGN4 supplementation [53, 54].

To further evaluate the systemic relevance of these gut-related changes, inflammatory markers were assessed. TNFα concentrations were significantly reduced following *B. bifidum* BGN4 supplementation, indicating an anti-inflammatory effect that may contribute to improved insulin sensitivity. Randomized controlled trials have shown that probiotic-induced reduction in inflammatory markers often parallel increases in beneficial genera, particularly *Bifidobacterium* and *Lactobacillus*, in overweight and obese adults [38, 55]. Although hs-CRP was defined as the primary outcome, no significant change was observed following the intervention. This may be attributed to the relatively low baseline inflammatory status of the participants, as well as the limited sensitivity of hs-CRP to detect subtle changes over short-term interventions in metabolically healthy individuals. Previous study has reported that shorter interventions tend to preferentially affect inflammatory cytokines and insulin sensitivity, whereas longer-term interventions are more likely to induce broader metabolic improvements, including reductions in body fat and hs-CRP levels [56].

We also analyzed markers related to insulin resistance to assess whether *B. bifidum* BGN4 supplementation could improve early indicators of metabolic dysfunction. Although participants were randomly assigned to each group, baseline differences in certain variables, including CRP and insulin, were observed, which can occur by chance in randomized trials, particularly with relatively small sample sizes. To minimize the potential bias arising from these baseline differences, intervention effects were primarily evaluated based on between-group comparisons of changes from baseline. Notably, supplementation with *B. bifidum* BGN4 significantly decreased fasting insulin concentrations, with trends toward improvement in fasting glucose and HOMA-IR. As fasting insulin is a recognized early predictor of metabolic disturbances in normoglycemic individuals, its reduction may indicate a preventive effect against the progression toward metabolic syndrome [57]. Similarly, baseline HOMA-IR was close to 2.9, the commonly used cut-off for identifying insulin resistance, and showed a decreasing trend following *B. bifidum* BGN4 supplementation [58]. Together, these findings suggest that BGN4 may exert insulin-lowering effects before the development of overt metabolic impairment.

In addition, Spearman’s correlation analysis revealed multiple associations between gut microbial taxa and metabolic and inflammatory biomarkers across all participants, supporting a potential link between overall microbial composition and host metabolic status. Among these, taxa that were significantly associated with glucose levels, including *Howardella*, *AAYW_s*, *Howardella ureilytica*, and *PAC001414_s*, belong to the family *Lachnospiraceae*. Previous studies have reported that *Lachnospiraceae* are positively associated with serum glucose levels, suggesting a potential link between this family and glucose metabolism [59, 60]. However, as these findings are based on family-level observations, further species-specific investigations are required to clarify their functional roles. In addition, the *Lactobacillus rogosae* group was positively associated with LDL-C levels in the present study, which is consistent with previous reports showing an increased abundance of this taxon in patients with non-alcoholic fatty liver disease, a condition characterized by elevated serum LDL-C levels [61, 62]. Furthermore, *Sellimonas intestinalis* was negatively associated with zonulin, suggesting a potential link with intestinal barrier function. This species has been proposed as a candidate biomarker of gut recovery and intestinal homeostasis, as its increased abundance has been observed following dysbiosis and is associated with the maintenance of intestinal barrier integrity [63]. Although several uncharacterized taxa, including *PAC000748_g*, *DQ456377_s*, and *EU460192_s*, showed significant correlations with metabolic markers in the present study, the biological relevance of these associations remains unclear due to the lack of functional characterization. Collectively, these findings suggest that the observed associations might reflect microbiome-host metabolic interactions at the community level.

Although the participants in this study exhibited body fat percentages above normal ranges, most baseline metabolic and inflammatory markers were within clinically normal limits. This phenotype corresponds to MHO or metabolically healthy overweight, who do not exhibit overt metabolic abnormalities despite excess adiposity [3]. MHO has been reported to be more prevalent in women and younger adults, consistent with the demographic characteristics of our study population [64], and has been estimated to account for approximately 15% of obese individuals in large-scale epidemiological studies [65]. However, accumulating evidence indicates that this phenotype is not metabolically stable, with a substantial proportion of individuals transitioning to metabolically unhealthy obesity (MUO) over time. Indeed, longitudinal studies have shown that more than 40% of individuals with MHO develop MUO during extended follow-up periods [66, 67]. In this context, the correlation analyses in the present study revealed positive associations between body weight and fasting glucose, insulin, HOMA-IR, and TNFα concentrations, as well as a significant association between TNFα and serum zonulin. 

This study represents the first randomized controlled trial to examine the effects of *B. bifidum* BGN4 in metabolically healthy adults with excessive adiposity. The strengths of the study include its rigorous double-blind, placebo-controlled design with stratified randomization and an adequately estimated sample size, which enhance methodological validity. Notably, improvements in inflammatory and metabolic markers were observed even among participants who were metabolically healthy at baseline, highlighting that probiotic intervention can exert beneficial effects prior to the onset of overt metabolic dysfunction. These findings suggest a potential preventive role of *B. bifidum* BGN4 by modulating early metabolic and inflammatory pathways associated with excessive adiposity.

Several limitations should also be acknowledged. First, the 8-week intervention period may have been insufficient to induce significant changes in body weight or fat mass. Second, the relatively young age of participants may have contributed to their normal baseline metabolic profiles, potentially limiting the detection of subclinical alterations. Third, lifestyle factors such as dietary intake were not strictly controlled, introducing possible confounding variables (Supplementary Figure S1). Finally, as a single-center study with a modest sample size, the generalizability of the findings remains limited and warrants confirmation in larger and metabolically diverse populations.

## Conclusion

Taken together, supplementation with *B. bifidum* BGN4 was associated with reduced serum zonulin and modest changes in TNFα and fasting insulin, in metabolically healthy adults with excessive adiposity. These findings suggest that modulation of gut permeability and microbiota composition might represent an early mechanistic pathway involved in metabolic processes prior to the onset of overt metabolic disease. While no changes in body weight or fat mass were observed over the 8-week intervention, the observed biomarker-level improvements highlight the beneficial role of strain-specific probiotic supplementation in individuals at risk of metabolic deterioration. Future studies in metabolically high-risk populations and with longer intervention periods, incorporating metagenomic and other multi-omics approaches, are warranted to further delineate strain-specific effects and host–microbe interactions and to clarify the clinical relevance of BGN4 supplementation.

## Supplementary Information

Additional file 1. Supplementary Fig. 1. Pearson correlations of anthropometric, metabolic and inflammatory parameters at baseline.Associations between insulin and body fat, HDL-cholesterol and body weight, and HOMA-IR and body weight.Relationships between triglyceridesand fasting insulin, HOMA-IR and fasting insulin, and TG and HOMA-IR.Associations of TNF-α with zonulin and fasting insulin.Strong positive correlation between LDL-cholesterol and total cholesterol. Linear regression lines with 95% confidence intervalsare shown. Pearson correlation coefficientsand p-values are presented in each panel

Additional file 2. Supplementary Fig. 2. Relative abundance of gut microbiota at thephylum andgenus levels at baseline and after 8 weeks in the placebo and probiotic groups. Taxa with a mean relative abundance of < 1% across all samples were grouped as “ETC.”Changes in α-diversity indicesat baseline and after 8 weeks in the placeboand probioticgroups. Data are presented as mean ± SD. Within-group comparisons between baseline and week 8 were performed using paired t-tests or Wilcoxon signed-rank tests, as appropriate. Between-group differences in changes from baseline were assessed using independent t-tests or Wilcoxon rank-sum tests

Additional file 3

## Data Availability

The datasets used and/or analysed during the current study are available from the corresponding author on reasonable request.

## Abbreviations

ALT: Alanine aminotransferase
AST: Aminotransferase
*B. bifidum*: *Bifidobacterium bifidum*
*B. **coprocola*: *Bifidobacterium* *coprocola*
CONSORT: Consolidated standards of reporting trials
DBP: Diastolic blood pressure
GI: Gastrointestinal
HDL-C: High-density lipoprotein cholesterol
HOMA-IR: Homeostasis model assessment of insulin resistance
hs-CRP: High-sensitivity C-reactive protein
LDA: Linear discriminant analysis
LDL-C: Low-density lipoprotein cholesterol
LEfSe: Linear discriminant analysis effect size
*L. plantarum*: *Lactiplantibacillus* *plantarum*
LPS: Lipopolysaccharides
MHO: Metabolically healthy obesity
MUO: Metabolically unhealthy obesity
qPCR: Quantitative polymerase chain reaction
SBP: Systolic blood pressure
SCFA: Short-chain fatty acid
SD: Standard deviation
SEM: Standard error of the mean
TAC: Total antioxidant capacity
TG: Triglycerides
TNF α: Tumor necrosis factor alpha
UV: Ultraviolet
