# Supplementary figures and images for: Probiotic Bifidobacterium bifidum BGN4 supplementation modulates gut microbiome composition and reduces circulating zonulin, TNFα, and insulin in adults with excess adiposity: a randomized, double-blind, placebo-controlled trial
Source: Nutr Metab (Lond). 2026 May 11;23:81. doi: 10.1186/s12986-026-01124-1 (PMC13361739; doi:10.1186/s12986-026-01124-1)

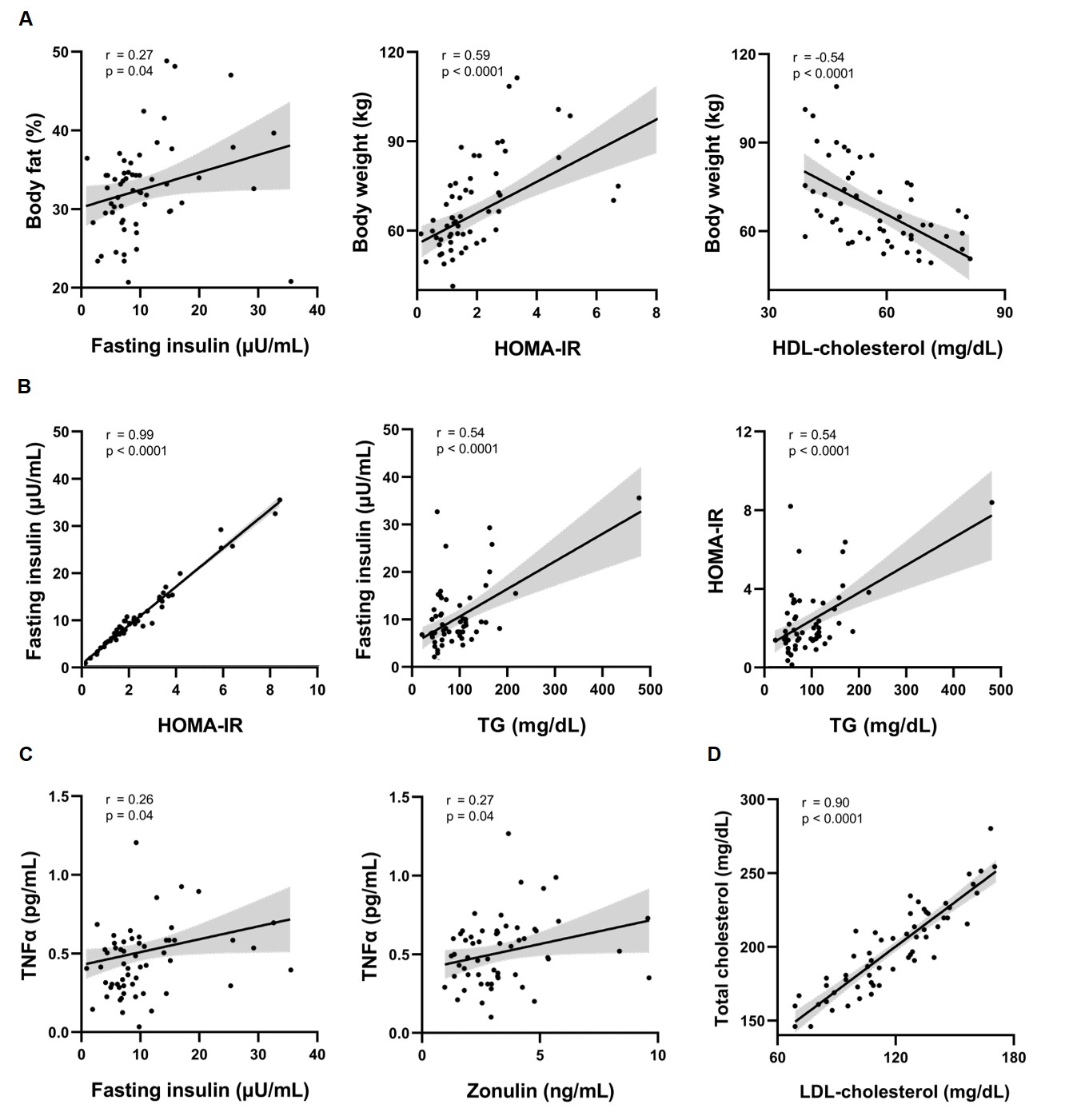

Supplement: Supplementary file 1 — Additional file 1. Supplementary Fig. 1. Pearson correlations of anthropometric, metabolic and inflammatory parameters at baseline.Associations between insulin and body fat, HDL-cholesterol and body weight, and HOMA-IR and body weight.Relationships between triglyceridesand fasting insulin, HOMA-IR and fasting insulin, and TG and HOMA-IR.Associations of TNF-α with zonulin and fasting insulin.Strong positive correlation between LDL-cholesterol and total cholesterol. Linear regression lines with 95% confidence intervalsare shown. Pearson correlation coefficientsand p-values are presented in each panel [file 12986_2026_1124_MOESM1_ESM.jpg]

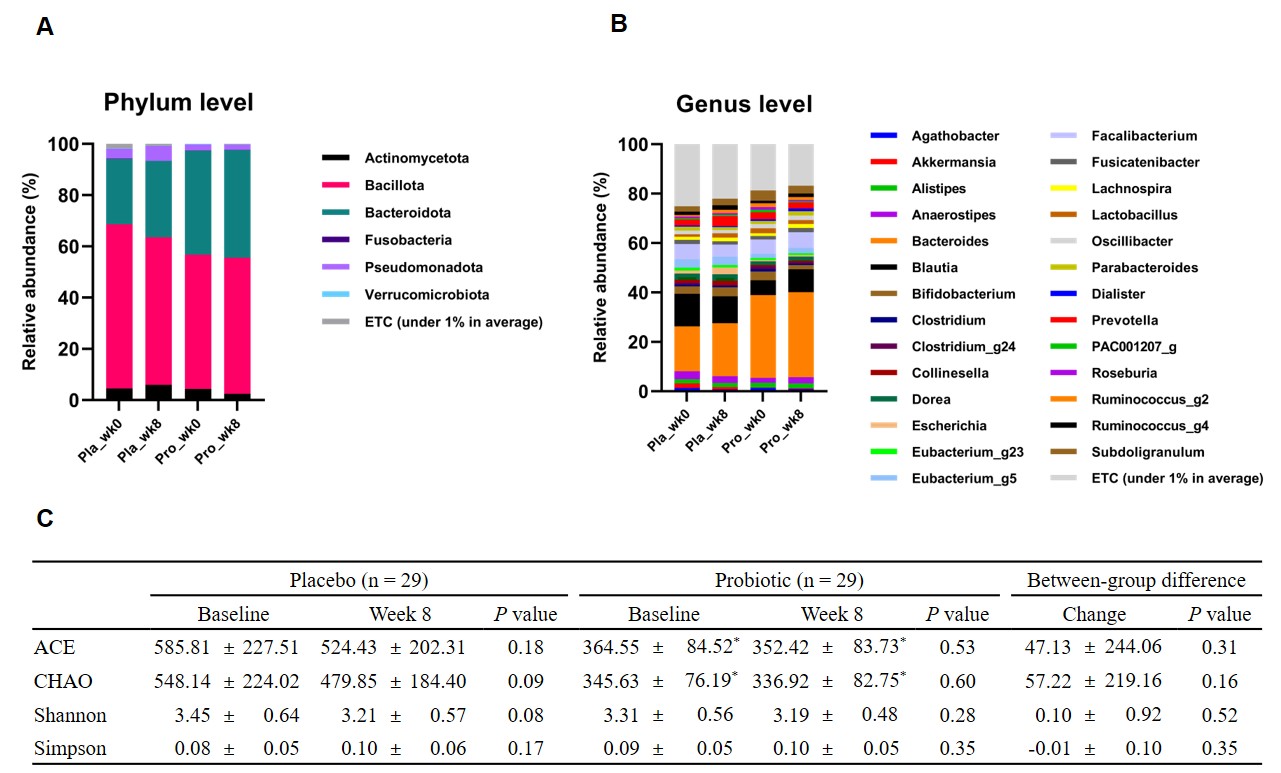

Supplement: Supplementary file 2 — Additional file 2. Supplementary Fig. 2. Relative abundance of gut microbiota at thephylum andgenus levels at baseline and after 8 weeks in the placebo and probiotic groups. Taxa with a mean relative abundance of < 1% across all samples were grouped as “ETC.”Changes in α-diversity indicesat baseline and after 8 weeks in the placeboand probioticgroups. Data are presented as mean ± SD. Within-group comparisons between baseline and week 8 were performed using paired t-tests or Wilcoxon signed-rank tests, as appropriate. Between-group differences in changes from baseline were assessed using independent t-tests or Wilcoxon rank-sum tests [file 12986_2026_1124_MOESM2_ESM.jpg]
