# Supplementary material for: Probiotic Bifidobacterium bifidum BGN4 supplementation modulates gut microbiome composition and reduces circulating zonulin, TNFα, and insulin in adults with excess adiposity: a randomized, double-blind, placebo-controlled trial
Source: Nutr Metab (Lond). 2026 May 11;23:81. doi: 10.1186/s12986-026-01124-1 (PMC13361739; doi:10.1186/s12986-026-01124-1)
Supplement: Supplementary file 3 — Additional file 3 [file 12986_2026_1124_MOESM3_ESM.docx]

**Supplementary Table 1. Relative abundance of gut microbiota at the phylum and genus levels at baseline and after 8 weeks in the placebo and probiotic groups**

|  | Placebo (n = 29) | | | | | | |  | Probiotic (n = 29) | | | | | | |  | Between-group difference | | | |
| --- | --- | --- | --- | --- | --- | --- | --- | --- | --- | --- | --- | --- | --- | --- | --- | --- | --- | --- | --- | --- |
|  | Baseline | | | Week 8 | | | *P* value ^2^ |  | Baseline | | | Week 8 | | | *P* value ^2^ |  | Change | | | *P* value ^3^ |
| p_*Actinomycetota* | 4.53 | ± | 6.00 | 5.75 | ± | 6.41 | 0.50 |  | 4.37 | ± | 9.26 | 2.43 | ± | 2.49 | 0.39 |  | -3.12 | ± | 10.35 | 0.17 |
| p_*Bacillota* | 63.99 | ± | 24.21 | 57.58 | ± | 24.14 | 0.24 |  | 52.06 | ± | 24.56 | 52.96 | ± | 39.43 | 0.89 |  | 7.09 | ± | 46.72 | 0.39 |
| p_*Bacteroidota* | 25.48 | ± | 24.29 | 29.84 | ± | 28.80 | 0.45 |  | 40.62 | ± | 27.69^*^ | 42.13 | ± | 30.70^*^ | 0.84 |  | -2.84 | ± | 9.53 | 0.77 |
| p_*Fusobacteria* | 0.13 | ± | 0.38 | 0.01 | ± | 0.02 | 0.13 |  | 0.07 | ± | 0.04 | 0.04 | ± | 0.07 | 0.81 |  | 0.09 | ± | 0.09 | 0.18 |
| p_*Pseudomonadota* | 3.89 | ± | 8.89 | 5.78 |  | 11.42 | 0.52 |  | 2.35 |  | 3.20 | 1.99 |  | 1.75 | 0.33 |  | -2.25 | ± | 1.52 | 0.98 |
| p_*Verrucomicrobiota* | 1.80 | ± | 5.17 | 0.94 |  | 3.23 | 0.67 |  | 0.20 |  | 0.57^*^ | 0.32 |  | 1.24 | 0.79 |  | 0.98 | ± | 0.78 | 0.69 |
| g_*Agathobacter* | 1.39 | ± | 2.19 | 0.75 | ± | 1.00 | 0.30 |  | 1.36 | ± | 1.54 | 0.99 | ± | 1.41 | 0.24 |  | 0.27 | ± | 0.48 | 0.57 |
| g_*Akkermansia* | 1.80 | ± | 5.07 | 0.93 | ± | 3.23 | 0.68 |  | 0.20 | ± | 0.57 | 0.32 | ± | 1.24 | 0.72 |  | 0.99 | ± | 0.78 | 0.55 |
| g_*Alistipes* | 1.68 | ± | 2.27 | 1.68 | ± | 2.85 | 0.18 |  | 1.86 | ± | 2.18 | 1.91 | ± | 2.22 | 0.94 |  | 0.04 | ± | 0.75 | 0.47 |
| g_*Anaerostipes* | 3.26 | ± | 4.08 | 2.63 | ± | 3.86 | 0.31 |  | 2.02 | ± | 2.41 | 2.52 | ± | 3.64 | 0.84 |  | 1.14 | ± | 1.04 | 0.41 |
| g_*Bacteroides* | 18.12 | ± | 20.47 | 21.49 | ± | 24.81 | 0.55 |  | 33.52 | ± | 25.86* | 34.36 | ± | 28.49* | 0.91 |  | -2.54 | ± | 8.85 | 0.64 |
| g_*Blautia* | 13.19 | ± | 15.70 | 10.86 | ± | 12.44 | 0.50 |  | 6.02 | ± | 6.63 | 9.24 | ± | 12.78 | 0.48 |  | 5.55 | ± | 3.77 | 0.33 |
| g_*Bifidobacterium* | 2.99 | ± | 4.69 | 3.7 | ± | 5.36 | 0.88 |  | 3.50 | ± | 8.59 | 1.76 | ± | 1.95 | 0.51 |  | -2.46 | ± | 1.85 | 0.41 |
| g_*Clostridium* | 0.77 | ± | 1.44 | 0.54 | ± | 1.47 | 0.19 |  | 0.89 | ± | 2.38 | 0.49 | ± | 1.11 | 0.52 |  | -0.26 | ± | 0.60 | 0.66 |
| g_*Clostridium_g24* | 0.74 | ± | 1.04 | 0.33 | ± | 0.33 | 0.06 |  | 1.03 | ± | 2.20 | 0.72 | ± | 1.04 | 0.94 |  | 0.11 | ± | 0.51 | 0.92 |
| g_*Collinsella* | 1.15 | ± | 1.92 | 1.85 | ± | 2.84 | 0.32 |  | 0.64 | ± | 0.94 | 0.48 | ± | 0.87 | 0.53 |  | -0.67 | ± | 0.38 | 0.17 |
| g_*Dorea* | 2.57 | ± | 3.10 | 2.67 | ± | 3.43 | 0.88 |  | 1.60 | ± | 1.89 | 1.72 | ± | 2.22 | 0.84 |  | 0.02 | ± | 0.77 | 0.77 |
| g_*Escherichia* | 1.24 | ± | 4.66 | 2.77 | ± | 7.79 | 0.66 |  | 0.54 | ± | 1.45 | 0.63 | ± | 1.00 | 0.11 |  | -1.44 | ± | 0.80 | 0.50 |
| g_*Escherichia_g23* | 1.12 | ± | 1.82 | 1.05 | ± | 1.99 | 0.88 |  | 0.80 | ± | 1.39 | 0.71 | ± | 1.17 | 0.55 |  | -0.02 | ± | 0.50 | 0.93 |
| g_*Escherichia_g5* | 3.40 | ± | 3.58 | 3.3 | ± | 4.63 | 0.89 |  | 1.65 | ± | 2.06* | 2.16 | ± | 2.74 | 0.47 |  | 0.61 | ± | 1.00 | 0.99 |
| g_*Faecalibacterium* | 6.17 | ± | 4.83 | 4.93 | ± | 5.37 | 0.32 |  | 5.84 | ± | 4.46 | 6.40 | ± | 6.74 | 0.29 |  | 1.80 | ± | 1.67 | 0.19 |
| g_*Fusicatenibacter* | 1.70 | ± | 2.92 | 1.34 | ± | 2.06 | 0.35 |  | 1.42 | ± | 1.85 | 1.69 | ± | 2.51 | 0.97 |  | 0.63 | ± | 0.67 | 0.58 |
| g_*Lachnospira* | 1.26 | ± | 2.29 | 1.49 | ± | 2.51 | 0.69 |  | 1.07 | ± | 2.10 | 1.62 | ± | 4.10 | 0.63 |  | 0.31 | ± | 1.08 | 0.60 |
| g_*Lactobacillus* | 0.95 | ± | 2.33 | 1.74 | ± | 7.97 | 0.27 |  | 1.99 | ± | 5.42 | 1.55 | ± | 5.27 | 0.99 |  | -1.24 | ± | 2.03 | 0.45 |
| g_*Oscillibacter* | 1.54 | ± | 2.56 | 1.32 | ± | 2.20 | 0.96 |  | 1.75 | ± | 2.49 | 1.89 | ± | 2.53 | 0.69 |  | 0.35 | ± | 0.65 | 0.87 |
| g_*Parabacteroides* | 1.57 | ± | 3.18 | 1.15 | ± | 1.48 | 0.88 |  | 1.27 | ± | 1.79 | 1.70 | ± | 2.56 | 0.63 |  | 0.85 | ± | 0.82 | 0.60 |
| g_*Dialister* | 0.53 | ± | 1.25 | 0.43 | ± | 0.80 | 0.74 |  | 0.69 | ± | 0.95 | 1.12 | ± | 1.67 | 0.34 |  | 0.53 | ± | 0.41 | 0.47 |
| g_*Prevotella* | 2.39 | ± | 5.03 | 4.01 | ± | 11.63 | 0.82 |  | 2.86 | ± | 7.62 | 2.46 | ± | 6.33 | 0.55 |  | -2.02 | ± | 2.74 | 0.57 |
| g_*PAC001207_g* | 0.48 | ± | 1.18 | 0.54 | ± | 1.71 | 0.48 |  | 1.00 | ± | 2.52 | 0.40 | ± | 0.76 | 0.20 |  | -0.66 | ± | 0.46 | 0.15 |
| g_*Roseburia* | 0.79 | ± | 0.98 | 0.70 | ± | 0.90 | 0.70 |  | 1.09 | ± | 1.27 | 0.58 | ± | 0.49 | 0.11 |  | -0.43 | ± | 0.33 | 0.40 |
| g_*Ruminococcus_g2* | 0.63 | ± | 0.84 | 1.35 | ± | 3.80 | 0.91 |  | 1.55 | ± | 2.64 | 1.23 | ± | 2.28 | 0.19 |  | -1.04 | ± | 0.80 | 0.38 |
| g_*Ruminococcus_g4* | 1.33 | ± | 2.29 | 1.33 | ± | 2.29 | 0.21 |  | 1.02 | ± | 1.24 | 1.36 | ± | 2.29 | 0.44 |  | -0.13 | ± | 0.58 | 0.12 |
| g_*Subdoligranulum* | 2.16 | ± | 1.87 | 2.69 | ± | 2.47 | 0.05 |  | 4.13 | ± | 4.68 | 3.20 | ± | 2.28 | 0.67 |  | -1.47 | ± | 0.95 | 0.48 |

^1^ Values are expressed as mean ± SD.

^2^ For within-group comparisons, paired-samples t-tests were employed for normally distributed data, while Wilcoxon signed-rank tests were utilized for skewed

data.

^3^ Differences between groups in changes from baseline were evaluated using independent-samples t-tests for normally distributed data and Wilcoxon rank-sum tests for skewed data. Asterisks (*) indicate significant differences between groups at the same time point, with *P* < 0.05.
